# Miscellany

**Published:** 1912-05

**Authors:** 


### MISCELLANY

Most of us have seen articles on the trade relations between Germany and the U. S., on account of potash, but few have realized that potassium is necessary in agriculture, beyond the minute amount normally present in the soil. Thus far, no considerable deposits of potash have been found in the U. S. except under such circumstances of dilution and presence of other materials as to make the cost of production prohibitive. Sea water contains about 10 ounces to the ton but the cost of separation would again be prohibitive. However, there has been discovered a natural means of condensation, in a gigantic sea weed which grows at a depth of 60 to 100 feet, abundantly along the Pacific coast. When air-dried, this yields 25 per cent. of potash salts and also 3 pounds of iodine per ton. The present problem therefore, is to devise some form of submarine harvesting machinery. (Abstract from Current Literature, February, 1912.)

---

Consumption of Sugar in the U. S. Last year, the continental U. S. consumed 7 billion, 670 million pounds of sugar, 81.75 pounds per capita, the maximum yet reached, being an increase from 72 pounds in 1901 and from 36.5 pounds in 1871. Some years ago, we found that the average consumption for about 40 dwellers in a residence hotel, was about 130 grams per capita per diem (less waste but plus an unknown and probably considerable quantity taken in the form of candy, soft drinks, extra meals, etc.), corresponding approximately to 100 pounds a year. From personal and observed experience, the conclusion was drawn that certain persons may take as much as 200 grams a day, corresponding to about 160 pounds a year, but this includes the natural sugar of fruit, etc. For the country as a whole, 100 pounds per capita per year, may be regarded as the ultimate maximum.

Saccharose, as is well known, is a double hexose derived from the maple, cane and beet. Originally—or rather aboriginally—the first was the chief source of sugar for what is now the northern states and it continued to be within the recollection of persons still living. Then, for many years, the sugar cane was almost the only commercial source. With the development of the beet sugar, industry, this last now furnishes about 2-3 of the domestic sugar.

In view of the recent agitation concerning the cost of living, it is interesting to note that the wholesale value of sugar is estimated at about 3 cents a pound and that $90 million worth of the sugar in 1911 came from foreign countries, $78 million worth from "possessions" and $90 million worth was raised in the states. A duty of $50 million was paid on imported sugar. It is not the province of a medical journal to enter the field of politics or even of political economy but in view of the paramount importance of maintaining a low standard of retail cost for food, the demonstration that sugar can be marketed at an ultimate price of 5 cents and that it has suddenly gone up to 6 or even 7 cents, apparently quite arbitrarily, there is a pretty obvious moral to be drawn.

---

Obstetrical Charts in colors, sent on receipt of 25c, postage paid. Ready for delivery June 1st. Battle & Co., St. Louis.

---

**Don't take them too seriously.**

Mrs. Fiske, in her recent play entitled Mrs. Bumpstead-Leigh, depicts the social ambition of the family of a patent medicine man. Whether we are to consider this as a play "with a purpose" or not, the fact remains that the essential vulgarity, ignorance and deception of the old style quack is impressed upon the audience during the comedy, in such a way as to educate more thoroughly than could be done by a campaign of serious literature and popular lectures.

Some of the strongest organizations of charlatans have been welded by the blows of denunciation whereas, if ignored or treated with not too contemptuous ridicule, they would have remained a heap of scraps that would have rusted out in a few years.

There is, in almost every phase of human activity, a minority that exists simply for the sake of being a minority; a cause that loves martyrdom more than what it nominally stands for; an under dog that turns its belly upward as naturally as does a dead fish. And there are men, not wise enough to occupy a high place in any sincere activity but shrewd enough to thrive by exploiting the fraternalism of a persecuted tenet.

Do you remember the old fashioned, picturesque, red shirted and helmeted volunteer firemen? Do you remember, as a little boy, being puzzled at the paradox of the double line pulling, by a white cord, the gaily painted hose cart, while two men at the pole of the cart resisted their efforts? And do you remember the kind grown up who explained that that little minority was what kept the cord from getting dirty and held the majority in even line? Don't worry about the men who are dragging against your professional efforts, as long as you are pulling the cart ahead. Let them alone, they are fulfilling a useful function. If you get out of line and drop the rope to kick off or to induce them to pull with you, you will spoil the whole procession.

---

Convalescence from the Exanthemata.—The first two or three months of the year are usually characterized, in the experience of the family physician, by the occurrence in his practice, of a crop of cases of the contagious diseases of children, especially scarlet fever, measles, German measle, etc. This is accounted for by the readiness with which contagion is spread in the schools, when ventilation of the school room is the least perfect and the closer housing of school children during school hours favors the distribution of communicable diseases. As the diseases in question are self-limited in nature, expectant and symptomatic treatment, together with precautions as to isolation, etc., is about all the physician is called upon to direct. It is well known, however, that in all but the mildest cases, the adolescent subject of scarlatina, or measles, is usually more or less debilitated or devitalized, when convalescence is established. Special care should be taken to avoid the administration of any tonic or reconstituent which is likely to disturb the child's digestion or, by inducing constipation, to minimize the appetite or desire for food.

Pepto-Mangan (Gude) is the ideal reconstructive tonic for these young patients, because it is pleasant to the taste, easily tolerable by the stomach and readily assimilable by blood and tissue and promptly efficient in restoring appetite, strength, color and general well-being.

